# Genital Immune Correlates of Seroprevalent and Seroincident *Herpes Simplex* Type 2 Infection Among Women Who Sell Sex in Nairobi, Kenya

**DOI:** 10.1111/aji.70273

**Published:** 2026-06-19

**Authors:** Suji Udayakumar, James Pollock, Erastus Irungu, Peter Muthoga, Wendy Adhiambo, Sanja Huibner, Mary Kungu'u, Rhoda Kabuti, Hellen Babu, Maisha Fiti Study Champions, Pauline Ngurukiri, Helen A. Weiss, Janet Seeley, Tanya Abramsky, Tara S. Beattie, Joshua Kimani, Rupert Kaul, Demtilla Gwala, Daisy Oside, Ruth Kamene, Agnes Watata, Agnes Atieno, Faith Njau, Elizabeth Njeri, Evelyn Orobi, Ibrahim Lwingi

**Affiliations:** ^1^ Department of Medicine University of Toronto Toronto Ontario Canada; ^2^ Department of Immunology University of Toronto Toronto Ontario Canada; ^3^ Partners For Health and Development in Africa (PHDA) Nairobi Kenya; ^4^ MRC International Statistics and Epidemiology Group, Department of Infectious Disease Epidemiology London School of Hygiene and Tropical Medicine, London Greater London UK; ^5^ Department of Global Health and Development London School of Hygiene and Tropical Medicine, London Greater London UK

**Keywords:** E‐cadherin, epithelial disruption, genital immunology, HIV prevention

## Abstract

**Problem:**

Most *Herpes simplex* virus type 2 (HSV‐2) infection is asymptomatic but increases the risk of HIV acquisition, possibly due to alterations in genital immunology. We examine associations of HSV‐2 prevalence and incidence with epithelial barrier disruption.

**Method of Study:**

The study was nested within the longitudinal Maisha Fiti cohort of women who sell sex in Nairobi, Kenya. HSV‐2 serostatus was assessed by Kalon HSV‐2 IgG assay. Socio‐behavioural characteristics were assessed by questionnaire and analysed by logistic regression. Immune factors (including soluble E‐cadherin (sE‐cad)) were assayed in cervicovaginal secretions by multiplex immunoassay, log‐transformed and analysed through linear regression.

**Results:**

Among 731 HIV‐negative participants, 414 (57%) were HSV‐2 seropositive. These women were older (median age 35 vs 28 years; *p* < 0.001) and reported increased intravaginal washing (64 vs 56%; *p* = 0.027) than those who were HSV‐2 seronegative. Genital sE‐cad levels were similar, and IL‐6 levels were lower in seropositive participants (1.15 vs 1.28 pg/mL, *p* < 0.01). Seroincidence was 10.7/100 person (95% CI: 7.3, 15.2) years among the 317 initially seronegative participants. Incident infection was associated with older age (31 vs 28 years, *p* = 0.001), increased number of clients (6 vs 4 clients/week, *p* = 0.005), and bacterial vaginosis (BV) (32 vs 15%, *p* = 0.009). Although women who acquired HSV‐2 had higher sE‐cad and lower MIP‐3*α* levels, there was no association after controlling for Nugent score.

**Conclusions:**

Subclinical epithelial barrier disruption is unlikely to be underpinning HIV acquisition in asymptomatic HSV‐2 infection. There was no evidence of genital immune predictors of HSV‐2 acquisition, whereas the vaginal microbiome is important.

## 1 Introduction

*Herpes simplex* virus type 2 (HSV‐2) infection is clinically asymptomatic in most individuals, but is associated with a three‐fold risk in human immunodeficiency virus (HIV) acquisition after controlling for behavioural parameters [1]. When symptomatic, HSV‐2 infection causes inflammatory anogenital ulcerations which are characterized by mucosal CD4+ T cell infiltration and mucosal epithelial barrier disruption [2, 3]. These alterations increase the density of HIV‐susceptible CD4+ T cells at the mucosal site of HIV exposure [4, 5] and enhance viral access to these target cells [6], respectively. Asymptomatic HSV‐2 infection does not (by definition) cause clinical ulceration, but is characterized by intermittent HSV‐2 reactivation and genital shedding [7], and an increased density of HIV‐susceptible CCR5/CD4+T cells in the endocervix and the foreskin [8, 9]. Genital inflammatory cytokines have not been found to be elevated in asymptomatic HSV‐2 infection [10], but it is unclear whether subclinical epithelial barrier disruption contributes to HIV susceptibility.

The probability of male‐female HSV‐2 transmission within serodiscordant couples is approximately 5%–20% annually, meaning that most sexual exposures do not lead to incident infection [11]. Though behavioral and biological factors that affect HSV‐2 susceptibility are established, such as condom use and bacterial vaginosis (BV) [12, 13], the genital immune correlates of HSV‐2 susceptibility have not been well described. One study found that HSV‐2 incident infection was linked to reduced preceding cervicovaginal levels of the inflammatory cytokines/chemokines interleukin (IL)‐6 and macrophage inflammatory protein (MIP)‐3*α* [14]. Furthermore, although HSV‐2 entry is also facilitated by epithelial disruption, it is unclear whether preceding subclinical epithelial barrier damage enhances HSV‐2 susceptibility.

Women who sell sex have high HIV seroprevalence and seroincidence, driven by behavioural and biological factors [15], particularly in sub‐Saharan Africa [16]. HSV‐2 is a lifelong infection with a seroprevalence that approaches 80% in women who sell sex [17], and is mostly asymptomatic in this population [18]. It has been estimated that the HIV Population Attributable Risk (PAR) of prevalent HSV‐2 infection is almost 50%, with an additional 4.5% attributable to incident HSV‐2 infection [19]. It is important to understand how HSV‐2 infection, and associated genital immune correlates, contribute to HIV susceptibility amongst HIV‐negative women who sell sex.

The objectives of the current study were to assess the association of seroprevalent and seroincident HSV‐2 infection with a panel of genital soluble immune factors in a large cohort of HIV‐uninfected women who sell sex in Nairobi, Kenya. This panel included soluble E‐cadherin (sE‐cad), a recently validated biomarker of epithelial barrier disruption that is elevated after minor endocervical trauma, and that is associated with immune markers of HIV susceptibility including inflammatory cytokines and an increased density of activated cervical CD4+ T cells [20, 21].

## 2 Methods

### 2.1 Study Design

This study was nested within the longitudinal Maisha Fiti study of women who sell sex in Nairobi, Kenya conducted from June 2019–March 2021. Exclusion criteria included current pregnancy, breast‐feeding or the presence of an underlying chronic illness.

The study design, process and data collection have previously been described in detail [22]. All participants provided written formal consent. The Maisha Fiti study was approved by the Kenyatta National Hospital Ethics and Research Committee (KNH ERC P778/11/2018), the London School of Hygiene and Tropical Medicine (LSHTM) Ethics Committee (Approval number: 16229) and the University of Toronto ethics committee (Approval number: 37046).

A detailed demographic and behavioural questionnaire was completed at enrolment, with collection of blood, urine and three vaginal swabs. Cervicovaginal secretions were self‐collected by participants using an Instead Softcup (Evofem, San Diego, CA), diluted 1:10 in phosphate buffered saline and cryopreserved at −80C. HIV status was determined through rapid test kits, with positive test results confirmed by HIV DNA Genexpert. Among HIV‐negative participants, blood was tested for *Treponema pallidum* (syphilis) infection, CRP levels and HSV‐2 infection. HSV‐2 serostatus was assessed using the Kalon HSV type 2 IgG assay according to manufacturer's instructions [23]. Values falling between calibrated negative and positive thresholds were recorded as “borderline.” Urine was collected for pregnancy testing, schistosomiasis diagnostics, and testing for sexually transmitted infections (STI) (i.e., *Chlamydia trachomatis* and *Neisseria gonorrhoeae*). Genital swabs were tested for BV via Nugent scoring and *Trichomonas vaginalis*.

### 2.2 Quantification of Soluble Immune Factors

Cervicovaginal secretions from self‐collected Softcup samples were assessed for the following soluble genital immune markers: sE‐cad, IL‐1*α*, IL‐1*β*, IL‐6, IL‐8, interferon‐inducible protein (IP‐10), monocyte chemoattractant protein‐1 (MCP‐1), monokine induced by interferon‐*γ* (MIG), MIP‐1*α*, MIP‐1*β*, MIP‐3*α*, matrix metalloproteinase (MMP)‐9 and tumour necrosis factor (TNF). Assays were performed in duplicate by electro‐chemiluminescence ELISA (Meso Scale Discovery [MSD], Rockville, MD). The lower limit of the detection range for the concentration of each immune marker was determined through the standard curves produced by the assay results. Samples with values within the detection range but a coefficient of variation exceeding 30% were repeated.

### 2.3 Statistical Analysis

Socio‐behavioural associations of HSV‐2 seropositivity and incidence were assessed by the chi‐square test (categorical variables) and Mann–Whitney U test (continuous variables). These comparisons were conducted using a weighted analysis to account for over‐sampling of younger women in the cohort.

Genital immune factor concentrations were log10‐transformed and compared between HSV‐2 seropositive and seronegative groups by Mann–Whitney U test. Linear regression models were constructed to explore the impact of HSV‐2 serostatus on genital immune parameter outcomes in multivariable analysis. The multivariable analysis controlled for socio‐behavioural variables identified a priori as potential confounders. These variables have been associated with HSV‐2 prevalence in literature (i.e., age, education, socio‐economic status (SES), BV, condom use, contraception, intravaginal washing, number of clients and bacterial STI) [12, 13, 18, 24, 25].

Prospective genital immune factor associations of HSV‐2 incidence among initially HSV‐2 seronegative participants were also assessed by Mann–Whitney U test. Associations were further investigated using multivariate linear regression controlling for a priori potential confounders as above. Statistical analysis was performed using R (version 4.2.1; Posit).

### 2.4 Exploratory Analysis

Socio‐behavioural associations of HSV‐2 seropositivity were explored using multivariable logistic regression to control for potential correlations between socio‐behavioural variables (i.e., age, age at sexual debut, number of clients, education, SES, condom use, contraception use, previous pregnancy, intravaginal washing, recent sexual assault, tobacco use, alcohol use, cannabis use, bacterial STI and BV). Odds ratios (OR) of association between each of the exposure variables and the serostatus were estimated using logistic regression.

Socio‐behavioural associations of HSV‐2 incidence were also explored using multivariable logistic regression to control for potential correlations between socio‐behavioural variables. This analysis controlled for fewer covariates than the previous analysis because of the small proportion of sero‐converters (i.e., age, age at sexual debut, number of clients, education, condom use, contraception use, previous pregnancy, intravaginal washing, recent sexual assault, bacterial STI and BV).

## 3 Results

### 3.1 Socio‐Behavioural Associations of Prevalent HSV‐2 Infection

The Maisha Fiti cohort consisted of 1003 women who sell sex in Nairobi, of whom 746 participants were HIV‐negative. HSV‐2 serology was performed for all 738 HIV‐negative participants who attended the baseline visit: Of these, 7 participants (0.95%) gave an indeterminate result and were excluded from subsequent analysis. Among the remaining 731 participants, 414 (57%) were HSV‐2 seropositive, with socio‐behavioural characteristics shown in Table 1. HSV‐2 seropositive participants were older (median: 35 versus 28 years, *p* < 0.001), less likely to have completed secondary education (27 vs 39%, *p* = 0.001), and more likely to report intravaginal washing (64 vs 56%, *p* = 0.027), than HSV‐2 seronegative participants.

**TABLE 1:** Socio‐behavioural characteristics of participant cohort.

Characteristic	HSV‐2 seropositive	HSV‐2 seronegative	*p*‐value
	(*n* = 414)	(*n* = 317)	
**Demographic (median, range)**			
Age	35 (19–45)	28 (18–45)	<0.001
Age at sexual debut	16 (6–25)	17 (2–26)	0.442
Number of clients	3 (0–60)	4 (0–70)	0.12
**Behavioural (*n*, %)**			
Completed secondary education	113 (27%)	123 (39%)	0.001
SES			
Lower^b^	122 (29%)	111 (35%)	0.262
Middle	137 (33%)	101 (32%)	
Upper	155 (37%)	106 (33%)	
Condom use	347 (84%)	264 (83%)	0.755
Contraception use to prevent pregnancy	350 (85%)	290 (91%)	0.004
Previous pregnancy	406 (98%)	295 (93%)	<0.001
Intravaginal practises (douching)	267 (64%)	178 (56%)	0.027
Sexual assault in last 7 days	30 (7%)	17 (5%)	0.272
Daily tobacco use^a^	162 (39%)	98 (31%)	0.686
Daily alcohol use^a^	85 (21%)	58 (18%)	0.637
Daily cannabis use^a^	30 (7%)	50 (16%)	<0.001
**Clinical (*n*, %)**			
Bacterial STI prevalence	42 (10%)	42 (13%)	0.197
BV	155 (37%)	116 (37%)	0.897

*Note:* Legend: HSV‐2 seropositive and seronegative groups were compared using the Chi‐square test for categorical variables and the Mann—Whitney U test for continuous variables.

^a^ indicates that substance use comparisons were conducted between frequency of use (never, once or twice, monthly, weekly vs daily/almost daily).

^b^ indicates that socioeconomic status (SES) comparison was conducted to see whether the distribution of Lower, Middle and Upper SES categories was different between the two serostatus groups. HSV‐2, Herpes simplex virus type 2; STI, sexually transmitted infections.

### 3.2 Soluble Genital Immune Associations of Prevalent HSV‐2 Infection

To determine whether prevalent HSV‐2 infection was associated with soluble genital immune factors, we initially compared levels of sE‐cad, IL‐1*α*, IL‐1*β*, IL‐6, IL‐8, IP‐10, MCP‐1, MIG, MIP‐1*α*, MIP‐1*β*, MIP‐3*α*, MMP‐9 and TNF between women who were HSV‐2 seropositive and those who were HSV‐2 seronegative in a bivariate analysis (Mann‐Whitney test). Cervicovaginal sE‐cad levels did not vary between HSV‐2 seropositive and seronegative groups (median concentration of 4.86 versus 4.84 pg/mL, *p* = 0.212). IL‐6 levels were lower in HSV‐2 seropositive participants as compared to seronegative participants (1.15 versus 1.28 pg/mL, *p* = 0.007). There was no evidence of differences in other genital immune markers based on HSV‐2 serostatus.

**FIGURE 1:**
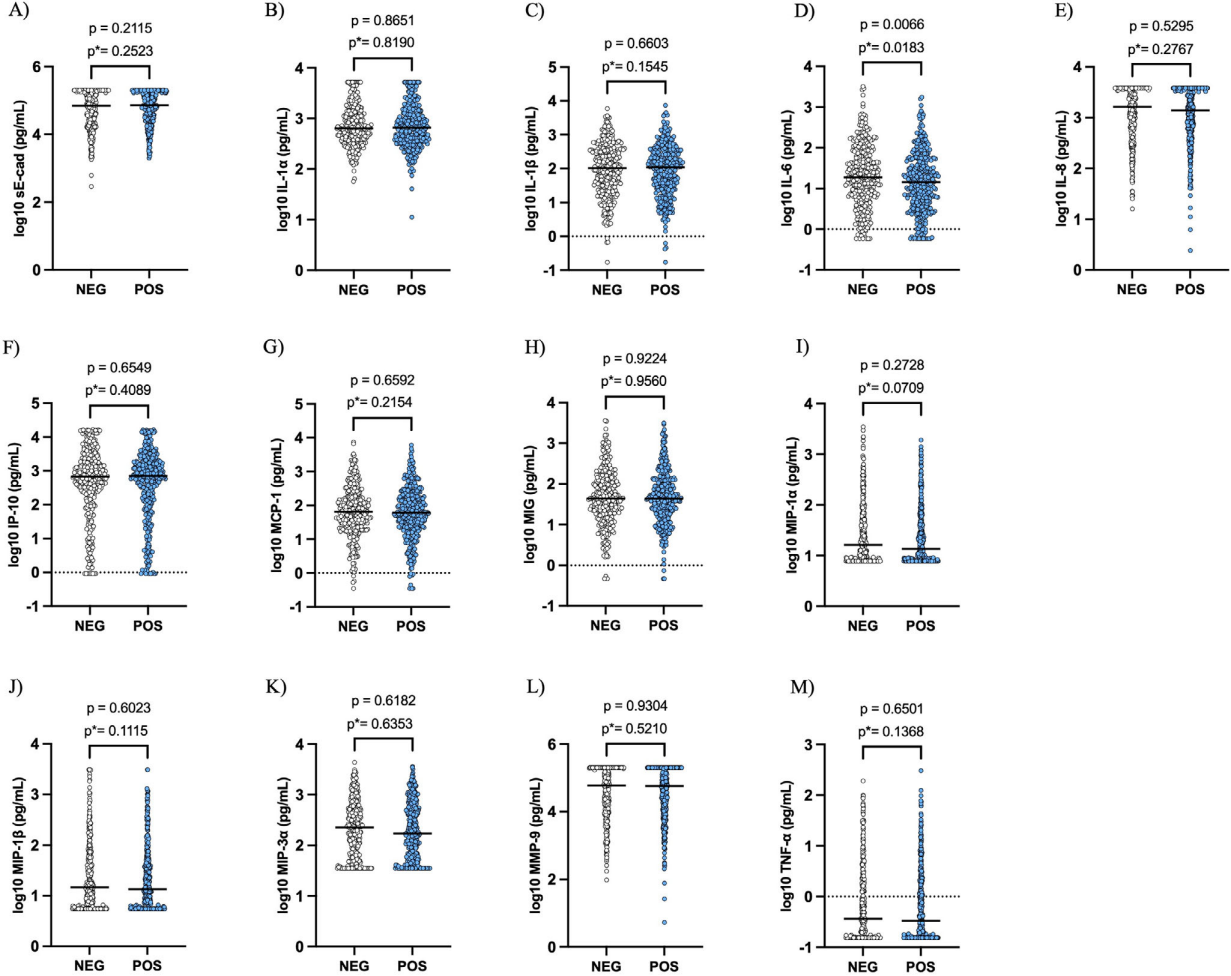
Prevalent HSV‐2 infection and genital immune marker concentrations. There was no association with HSV‐2 serostatus and the evaluated immune markers (A‐C, E‐M), except for IL‐6 (D). Results for Mann‐Whitney U Test and p‐values for each pair‐wise comparison are indicated above the graph. *p*‐value with an asterisk indicates multivariable regression results. Solid line indicates the median. Dashed line indicates 0 pg/mL. sE‐cad, soluble e‐cadherin; IL, interleukin; IP, interferon‐inducible protein; MCP, monocyte chemoattractant protein, MIG, monokine induced by interferon‐*γ*; MIP, macrophage inflammatory protein; MMP, matrix metalloproteinase; NEG, negative; POS, positive; TNF, tumour necrosis factor.

We then explored immune associations of prevalent HSV‐2 infection using multivariable linear regression analysis that controlled for variables reported to be associated with HSV‐2 infection (i.e., age, education, SES, BV, condom use, contraception, intravaginal washing, number of clients and bacterial STI). Multivariable models showed no evidence of association between vaginal sE‐cad levels and HSV‐2 serostatus (*β* = 0.042, *p* = 0.252). HSV‐2 infection remained associated with decreased vaginal IL‐6 levels (*β* = −0.148, *p* = 0.018) (Figure 1).

### 3.3 Socio‐Behavioural Associations of Incident HSV‐2 Infection

Of the 317 Maisha Fiti participants who were HSV‐2 seronegative at baseline, 31 (10%) seroconverted during follow‐up. The annual seroincidence was 10.7 per 100 person years (95% CI: 7.3, 15.2). Participants who acquired HSV‐2 infection during the course of the study were older (median age = 31 vs 28 years, *p* = 0.001), had a higher number of clients in the past week (median = 6 vs 4 clients, *p* = 0.005), and were more likely to have BV at baseline (32 vs 15%, *p* = 0.009) compared to those who remained HSV‐2 seronegative (Table 2).

**TABLE 2:** Socio‐behavioural characteristics of incident HSV‐2 infection.

	Remained seronegative	Seroconverter	
Characteristic	(*n* = 286)	(*n* = 31)	*p*‐value
**Demographic (median, range)**			
Age	28 (18–45)	31 (19–44)	0.001
Age at sexual debut	17 (2–26)	16 (9–25)	0.397
Number of clients	4 (0–70)	6 (0–35)	0.005
**Behavioural (*n*, %)**			
Completed secondary education	107 (37%)	16 (52%)	0.14
SES			
Lower^b^	100 (35%)	11 (35%)	0.251
Middle	94 (33%)	6 (19%)	
Upper	93 (32%)	14 (45%)	
Condom use	236 (83%)	27 (87%)	0.34
Contraception use to prevent pregnancy	261 (91%)	29 (94%)	0.81
Previous pregnancy	264 (92%)	30 (97%)	0.213
Intravaginal practises (douching)	160 (56%)	14 (45%)	0.227
Sexual assault in last 7 days	13 (5%)	3 (10%)	0.29
Daily tobacco use^a^	84 (29%)	16 (52%)	0.491
Daily alcohol use^a^	48 (17%)	10 (32%)	0.134
Daily cannabis use^a^	46 (16%)	4 (13%)	0.86
**Clinical (*n*, %)**			
Bacterial STI prevalence	37 (13%)	4 (13%)	0.98
BV	42 (15%)	10 (32%)	0.009

*Note:* Legend: Participants who remained seronegative and participants who seroconverted during the study period were compared using the Chi‐square test for categorical variables and the Mann–Whitney U test for continuous variables.

Abbreviations: HSV‐2, Herpes simplex virus type 2; STI, sexually transmitted infections.

^a^ indicates that substance use comparisons were conducted between frequency of use (never, once or twice, monthly, weekly vs daily/almost daily).

^b^ indicates that socioeconomic status (SES) comparison was conducted to see whether the distribution of Lower, Middle and Upper SES categories was different between the two groups.

### 3.4 Genital Immune Predictors of Incident HSV‐2 Infection

To characterize potential immune associations of increased HSV‐2 susceptibility, we compared baseline levels of sE‐cad, IL‐1*α*, IL‐1*β*, IL‐6, IL‐8, IP‐10, MCP‐1, MIG, MIP‐1*α*, MIP‐1*β*, MIP‐3*α*, MMP‐9 and TNF between women who acquired HSV‐2 and those who remained sero‐negative. In a bivariate analysis, seroconverters had higher baseline levels of cervicovaginal sE‐cad (median concentration of 5.14 vs 4.79 pg/mL, *p* = 0.005) and lower levels of MIP‐3*α* (2.05 vs 2.36 pg/mL, *p* = 0.039); levels of other soluble immune did not vary between groups (*p* > 0.05).

**FIGURE 2:**
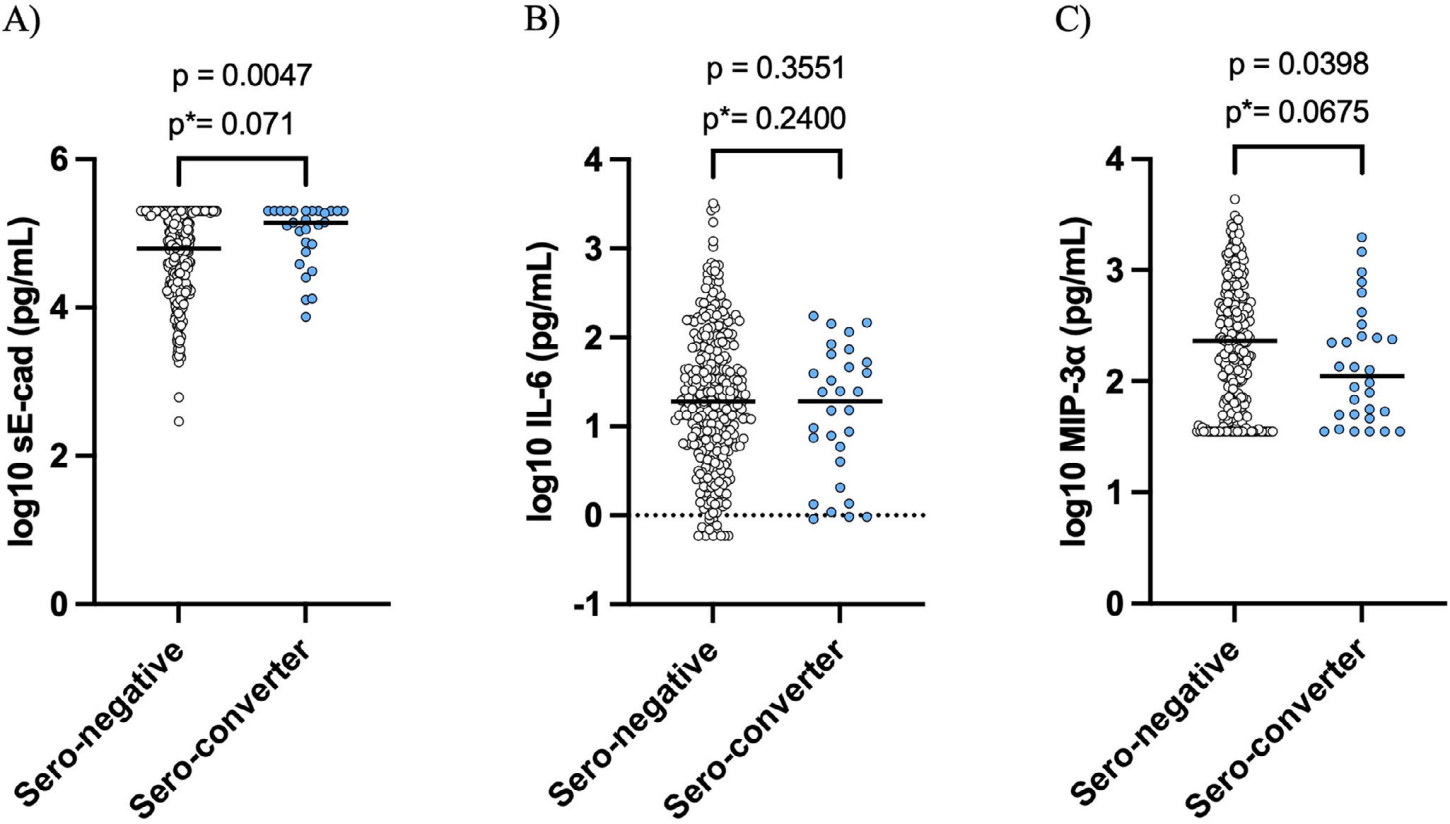
Incident HSV‐2 infection and genital immune marker concentrations. There was an association with incident HSV‐2 infection sE‐cad (A) and MIP‐3*α* (C). Results for Mann‐Whitney U Test and *p*‐values for each pair‐wise comparison are indicated above the graph. *p*‐value with an asterisk indicates multivariate comparison results. Solid line indicates the median. Dashed line indicates 0 pg/mL. sE‐cad, soluble e‐cadherin; IL, interleukin; MIP, macrophage inflammatory protein.

In multivariable linear regression analysis that controlled for variables associated with HSV‐2 infection (i.e., age, education, SES, BV, condom use, contraception, intravaginal washing, number of clients and bacterial STI), the associations of incident HSV‐2 infection with sE‐cad and MIP‐3*α* were weakened (Figure 2). BV Nugent score was independently associated with levels of sE‐cad (*β* = 0.11, *p* < 0.001) (Table S1).

### 3.5 Exploratory Analysis of Socio‐Behavioral Associations of HSV‐2 Serostatus and Incidence

Socio‐behavioral differences in HSV‐2 seroprevalent and incident groups were further explored using multivariable logistic regression. HSV‐2 seropositive participants were older (OR = 1.15, *p* < 0.001), more likely to be of a higher SES (OR = 2.60, *p* = 0.031) and more likely to report intravaginal washing (OR = 2.18, *p* = 0.021) than HSV‐2 seronegative participants (Table S2).

Participants who acquired HSV‐2 infection during the course of the study were older (OR = 1.09, *p* < 0.01) and were more likely to complete secondary education (OR = 3.34, *p* = 0.006) compared to those who remained HSV‐2 seronegative. There was some evidence that sero‐converters were more likely to have BV at baseline (OR = 1.58 *p* = 0.053) (Table S3).

## 4 Discussion

When symptomatic, HSV‐2 infection is characterized by genital ulcerations that disrupt mucosal integrity and dermal infiltration by high numbers of activated CD4+ T cells [2, 3], both of which may contribute to enhanced HIV susceptibility [26]. In addition, although most people with HSV‐2 infection, including female sex workers from Nairobi, have no history of genital ulceration [18, 27], HIV susceptibility remains substantially enhanced even in the context of asymptomatic HSV‐2 infection [8]. Prior small studies have demonstrated that asymptomatic HSV‐2 infection is associated with an increased density of HIV‐susceptible target cells in the endocervix [8], but generally not with increased pro‐inflammatory cytokines in cervicovaginal secretions [10]. In the current study we aimed to characterize the associations of soluble genital immune factors with both prevalent and incident HSV‐2 infection in a large cohort of women who sell sex in Nairobi, Kenya, and to assess‐for the first time, to our knowledge‐potential associations of HSV‐2 infection with vaginal levels of soluble E‐cadherin, a biomarker of epithelial disruption.

After controlling for potential confounders such as BV, which causes epithelial disruption and has been linked to HSV‐2 [13, 28], we find no soluble immune associations of seroprevalent HSV‐2 infection. This suggests that subclinical epithelial barrier disruption is not a major mechanism underpinning increased HIV susceptibility in HSV‐2 seropositive individuals, though studies have not directly evaluated the association of elevated soluble e‐cadherin with HIV acquisition.

There were no differences in levels of other genital immune markers between our groups, except for IL‐6, which was significantly higher in those who were HSV‐2 seronegative. Though this is contrary to previous findings where no proinflammatory cytokines were associated with prevalent HSV‐2 infection [10], it has been suggested that mucosal upregulation of IL‐6 may be protective against acquisition of HSV‐2 infection [14].

An association between HSV‐2 incident infection and lower cervicovaginal levels of inflammatory cytokines/chemokines, specifically IL‐6, has previously been reported [14]. In our analysis of prospective genital immune markers of HSV‐2 incident infection we found no association between HSV‐2 incidence and IL‐6 levels. Furthermore, although seroconverters had increased sE‐cad and decreased MIP‐3*α* at baseline, these associations were lost when controlling for the Nugent score, suggesting that the immune associations may be confounded by BV, a condition that both increases HSV‐2 susceptibility [13] and is associated with epithelial disruption and altered vaginal cytokines [29]. Therefore, our analysis does not suggest that inflammatory cytokines/chemokine levels in vaginal secretions play a major role in HSV‐2 protection, or that preceding epithelial barrier disruption facilitates HSV‐2 acquisition. These findings have important implications for HIV acquisition in populations with an increased prevalence of BV and/or HSV‐2, both of which have been demonstrated in Black women from sub‐Saharan Africa and North America [30, 31].

Our analysis has limitations and strengths. As the time between baseline and follow‐up visits for seroconverters ranged from 2 to 17 months, it is uncertain whether participants may have been infected with HSV‐2 at the baseline visit but not yet have seroconverted. We do not have details on whether participants had symptomatic or asymptomatic HSV‐2 infection, or whether they had HSV‐2 reactivation and shedding because we did not collect data on prior or current genital symptoms, as all participants were already accessing HIV/STI care through the affiliated Sex Worker Outreach Program (SWOP) Clinics. However, as we did not see differences in levels of sE‐cad between participants who were HSV‐2 seropositive and seronegative in our study, we can conclude that HSV‐2 infection is not associated with epithelial barrier disruption. In addition, levels of sE‐cad were above the level of quantification for 146 participants in our cohort, limiting our ability to study participants with high levels of epithelial barrier disruption. We did not have access to cytobrush or biopsies, and so could not perform immunohistochemistry or assess cellular markers. All participants in our study were female sex workers, and sex work may alter genital immunology in several different ways, and it would be important to confirm our findings in lower risk populations. However, our large sample size and the inclusion of analyses adjusted for potential confounders increases the reliability and generalizability of our findings. In addition, the inclusion of a prospective analysis on HSV‐2 incidence enhances the clinical relevance of the study's conclusions.

In summary, we demonstrate that epithelial barrier disruption is not likely to be a major mechanism underpinning increased HIV acquisition among individuals with asymptomatic HSV‐2 infection. We also see that genital epithelial barrier disruption and immune markers are not predictive of increased HSV‐2 susceptibility, whereas the microbiota has an important role. Future research should focus on further investigating the relationships between HSV‐2 infection, BV, and HIV.

## Ethics Statement

The authors confirm that the ethical policies of the journal, as noted on the journal's author guidelines page, have been adhered to. All participants provided written formal consent. The Maisha Fiti study was approved by the Kenyatta National Hospital Ethics and Research Committee (KNH ERC P778/11/2018), the London School of Hygiene and Tropical Medicine (LSHTM) Ethics Committee (Approval number: 16229) and the University of Toronto ethics committee (Approval number: 37046).

## Conflicts of Interest

The authors declare no competing interests.

## Supporting information




**Supporting Information**: aji70273‐sup‐0001‐SuppMat.docx
