# Supplementary material for: Genital Immune Correlates of Seroprevalent and Seroincident Herpes Simplex Type 2 Infection Among Women Who Sell Sex in Nairobi, Kenya
Source: Am J Reprod Immunol. 2026 Jun 19;95(6):e70273. doi: 10.1111/aji.70273 (PMC13281368; doi:10.1111/aji.70273)
Supplement: Supplementary file 1 — Supporting Information: aji70273‐sup‐0001‐SuppMat.docx [file AJI-95-e70273-s001.docx]

**Supplementary Table 1. Incident HSV-2 infection and soluble E-cadherin** **concentrations.**

| **Variable** | **Estimate** | **Standard error** | **T- statistic** | **p-value** |
| --- | --- | --- | --- | --- |
| HSV-2 Seroconversion | 0.18 | 0.10 | 1.82 | 0.071 |
| Age | 0.00 | 0.00 | -0.01 | 0.996 |
| Number of clients | 0.00 | 0.00 | 0.29 | 0.77 |
| Completed secondary education | -0.08 | 0.06 | -1.42 | 0.157 |
| Socio-economic status (Middle) | 0.07 | 0.06 | 1.15 | 0.252 |
| Socio-economic status (Upper) | 0.06 | 0.06 | 0.89 | 0.374 |
| Condom use | -0.02 | 0.07 | -0.36 | 0.719 |
| Contraception use to prevent pregnancy | 0.15 | 0.09 | 1.71 | 0.089 |
| Intravaginal practises (douching) | 0.06 | 0.05 | 1.07 | 0.287 |
| Bacterial STI prevalence | 0.07 | 0.07 | 1.00 | 0.317 |
| Bacterial vaginosis Nugent score | 0.11 | 0.01 | 12.31 | <0.001 |

Legend: Prospective associations between HSV-2 incidence and soluble E-cadherin (sE-cad) concentrations were assessed using multivariate linear regression controlling for socio-behavioural factors. ** indicates that socio-economic status (SES) were conducted between Lower SES and either Upper or Middle SES. Abbreviations: HSV-2, Herpes simplex virus type 2; STI, sexually transmitted infections; OR, odds ratio; LL, lower limit; UL, upper limit.

**Supplementary Table 2. Exploratory analysis of socio-behavioural associations of HSV-2 prevalence in the participant cohort.**

| **Characteristic** | **HSV-2 seropositive** | **HSV-2 seronegative** | **Logistic regression analysis** | | | |
| --- | --- | --- | --- | --- | --- | --- |
|  | **(n = 414)** | **(n = 317)** | **OR** | **95% LL** | **95% UL** | **p-value** |
| **Demographic (median, range)** |  |  |  |  |  |  |
| Age | 35 (19-45) | 28 (18-45) | 1.15 | 1.09 | 1.22 | <0.001 |
| Age at sexual debut | 16 (6-25) | 17 (2-26) | 1.02 | 0.90 | 1.17 | 0.744 |
| Number of clients | 3 (0-60) | 4 (0-70) | 0.99 | 0.93 | 1.05 | 0.648 |
| **Behavioural (n, %)** |  |  |  |  |  |  |
| Completed secondary education | 113 (27%) | 123 (39%) | 0.47 | 0.22 | 1.00 | 0.053 |
| Socio-economic status |  |  |  |  |  |  |
| Lower | 122 (29%) | 111 (35%) |  |  |  |  |
| Middle** | 137 (33%) | 101 (32%) | 2.10 | 0.94 | 4.80 | 0.074 |
| Upper** | 155 (37%) | 106 (33%) | 2.60 | 1.10 | 6.32 | 0.031 |
| Condom use | 347 (84%) | 264 (83%) | 0.92 | 0.34 | 2.46 | 0.866 |
| Contraception use to prevent pregnancy | 350 (85%) | 290 (91%) | 0.48 | 0.12 | 1.75 | 0.28 |
| Previous pregnancy | 406 (98%) | 295 (93%) | 3.41 | 0.47 | 70.45 | 0.292 |
| Intravaginal practises (douching) | 267 (64%) | 178 (56%) | 2.18 | 1.13 | 4.26 | 0.021 |
| Sexual assault in last 7 days | 30 (7%) | 17 (5%) | 2.05 | 0.61 | 7.59 | 0.259 |
| Daily tobacco use* | 162 (39%) | 98 (31%) | 1.31 | 0.58 | 3.00 | 0.514 |
| Daily alcohol use* | 85 (21%) | 58 (18%) | 0.63 | 0.18 | 2.11 | 0.455 |
| Daily cannabis use* | 30 (7%) | 50 (16%) | 0.73 | 0.31 | 1.72 | 0.474 |
| **Clinical (n, %)** |  |  |  |  |  |  |
| Bacterial STI prevalence | 42 (10%) | 42 (13%) | 1.50 | 0.53 | 4.29 | 0.443 |
| Bacterial vaginosis | 155 (37%) | 116 (37%) | 0.88 | 0.35 | 2.18 | 0.782 |

Legend: HSV-2 seropositive and seronegative groups were compared logistic regression analysis, with sero-status as the outcome. * indicates that substance use comparisons were conducted between frequency of use (never, once or twice, monthly, weekly vs daily/almost daily). ** indicates that socio-economic status (SES) were conducted between Lower SES and either Upper or Middle SES. Abbreviations: HSV-2, Herpes simplex virus type 2; STI, sexually transmitted infections; OR, odds ratio; LL, lower limit; UL, upper limit.

**Supplementary Table 3. Exploratory analysis of socio-behavioural associations of HSV-2 incidence in the participant cohort.**

| **Characteristic** | **Remained seronegative** | **Seroconverter** | **Logistic regression analysis** | | | |
| --- | --- | --- | --- | --- | --- | --- |
|  | **(n = 286)** | **(n = 31)** | **OR** | **95% LL** | **95% UL** | **p-value** |
| **Demographic (median, range)** |  |  |  |  |  |  |
| Age | 28 (18-45) | 31 (19-44) | 1.09 | 1.03 | 1.16 | 0.004 |
| Number of clients | 4 (0-70) | 6 (0-35) | 1.03 | 0.98 | 1.07 | 0.16 |
| **Behavioural (n, %)** |  |  |  |  |  |  |
| Completed secondary education | 107 (37%) | 16 (52%) | 3.34 | 1.43 | 8.16 | 0.006 |
| Condom use | 236 (83%) | 27 (87%) | 3.21 | 0.87 | 20.82 | 0.13 |
| Contraception use to prevent pregnancy | 261 (91%) | 29 (94%) | 1.80 | 0.45 | 12.27 | 0.467 |
| Previous pregnancy | 289 (90%) | 28 (93%) | 0.83 | 0.19 | 5.78 | 0.82 |
| Intravaginal practises (douching) | 160 (56%) | 14 (45%) | 0.73 | 0.31 | 1.70 | 0.468 |
| Sexual assault in last 7 days | 13 (5%) | 3 (10%) | 3.42 | 0.70 | 13.07 | 0.089 |
| **Clinical (n, %)** |  |  |  |  |  |  |
| Bacterial STI prevalence | 37 (13%) | 4 (13%) | 1.05 | 0.28 | 3.09 | 0.94 |
| Bacterial vaginosis | 42 (15%) | 10 (32%) | 1.58 | 1.00 | 2.53 | 0.053 |

Legend: Participants who remained seronegative and participants who seroconverted during the study period were compared through logistic regression analysis, with sero-conversion as the outcome. HSV-2, Herpes simplex virus type 2; STI, sexually transmitted infections; OR, odds ratio; LL, lower limit; UL, upper limit.
